# Association between serum oestradiol level on the hCG administration day and neonatal birthweight after IVF-ET among 3659 singleton live births

**DOI:** 10.1038/s41598-021-85692-7

**Published:** 2021-03-16

**Authors:** Yu Liu, Jing Li, Wanyu Zhang, Yihong Guo

**Affiliations:** Reproductive Medicine Center, The First Affiliated Hospital of Zhengzhou University, Zhengzhou, People’s Republic of China

**Keywords:** Diseases, Medical research, Signs and symptoms

## Abstract

Oestradiol, an important hormone in follicular development and endometrial receptivity, is closely related to clinical outcomes of fresh in vitro fertilization-embryo transfer (IVF-ET) cycles. A supraphysiologic E2 level is inevitable during controlled ovarian hyper-stimulation (COH), and its effect on the outcome of IVF-ET is controversial. The aim of this retrospective study is to evaluate the association between elevated serum oestradiol (E2) levels on the day of human chorionic gonadotrophin (hCG) administration and neonatal birthweight after IVF-ET cycles. The data of 3659 infertile patients with fresh IVF-ET cycles were analysed retrospectively between August 2009 and February 2017 in First Hospital of Zhengzhou University. Patients were categorized by serum E2 levels on the day of hCG administration into six groups: group 1 (serum E_2_ levels ≤ 1000 pg/mL, n = 230), group 2 (serum E2 levels between 1001 and 2000 pg/mL, n = 524), group 3 (serum E_2_ levels between 2001 and 3000 pg/mL, n = 783), group 4 (serum E_2_ levels between 3001 and 4000 pg/mL, n = 721), group 5 (serum E2 levels between 4001 and 5000 pg/mL, n = 548 ), and group 6 (serum E2 levels > 5000 pg/mL, n = 852). Univariate linear regression was used to evaluate the independent correlation between each factor and outcome index. Multiple logistic regression was used to adjust for confounding factors. The LBW rates were as follows: 3.0% (group 1), 2.9% (group 2), 1.9% (group 3), 2.9% (group 4), 2.9% (group 5), and 2.0% (group 6) (*P* = 0.629), respectively. There were no statistically significant differences in the incidences of neonatal LBW among the six groups. We did not detect an association between peak serum E2 level during ovarian stimulation and neonatal birthweight after IVF-ET. The results of this retrospective cohort study showed that serum E2 peak levels during ovarian stimulation were not associated with birth weight during IVF cycles. In addition, no association was found between higher E2 levels and increased LBW risk. Our observations suggest that the hyper-oestrogenic milieu during COS does not seem to have adverse effects on the birthweight of offspring after IVF. Although this study provides some reference, the obstetric-related factors were not included due to historical reasons. The impact of the high estrogen environment during COS on the birth weight of IVF offspring still needs future research.

## Background

Since the birth of Louise Brown in the UK in 1978^1^, the use of in vitro fertilization (IVF) to treat infertility has steadily increased, with more than seven million children born worldwide^2^. In vitro fertilization-embryo transfer (IVF-ET) is the main component of assisted reproductive technology (ART) and the most effective method to help infertile patients conceive. However, compared with infants conceived naturally, IVF-conceived infants, whether in a multiple or singleton pregnancy, usually have a higher risk of adverse perinatal outcomes, including low birth weight (LBW) and small for gestational age (SGA)^3^. It is still unclear whether these increased risks are due to the inherent characteristics of infertility, the IVF treatment itself, or a combination of both^4^. Controlled ovarian hyper-stimulation (COH) is considered a key determinant of IVF success, wherein multiple dominant follicles are recruited to increase the number of eggs for harvest in a single cycle. During the COH cycle, serum oestradiol (E2) levels can be increased more than tenfold that of a natural cycle. However, recent studies suggest that reproductive physiological E2 levels during COH may produce an unsatisfactory peri-implantation uterine environment, which leads to placental abnormalities and, ultimately, to adverse neonatal outcomes, such as preeclampsia, LBW, and SGA^3^. However, the effects of exposure to such high E2 levels on the day of human chorionic gonadotropin (hCG) injection during the IVF cycle are still unclear^5^. Recent evidence suggests that serum E2 has a concentration-dependent effect on pregnancy and birth rates^5^. However, Zavy et al.^6^ and Wang et al.^7^ reported that serum E2 on the day of hCG administration does not change the pregnancy rate. Based on these data, the importance of managing high E2 levels on the day of hCG administration remains controversial in terms of IVF outcomes. The objective of our study was to evaluate the effect of the hCG-day serum E2 level on neonatal birthweight after IVF-ET with COH.

## Methods

### Ethical approval

This study was approved by the Institutional Ethics Committee of the First Hospital of Zhengzhou University. All patients signed informed consent forms. All methods were performed in accordance with the relevant guidelines and regulations.

### Patient selection

This was a single-centre retrospective cohort study. Patients who were undergoing IVF with gonadotropin and a GnRH agonist for COH were enrolled from August 2009 to February 2017. Patients were excluded from the study if they experienced one of the following conditions: (1) moderate or severe ovarian hyper-stimulation syndrome (OHSS) during COH; (2) pregnancy complications including gestational diabetes mellitus, hypertensive disorders and thyroid diseases; (3) vanishing twin syndrome, defined as a foetus with cardiac activity plus one or more gestational sacs or a foetus without cardiac activity^8^; or (4) loss to follow-up or loss of core data in our electronic database (e.g., E2 level on the trigger day).

In cases where a patient has given birth more than once, only the first live birth was included in the analysis during the study period. Ultimately, 3659 patients constituted our final study cohort. According to serum E2 levels on the day of hCG administration, the patients were categorized into six groups: group 1 (serum E_2_ levels ≤ 1000 pg/mL, n = 230), group 2 (serum E_2_ levels 1001–2000 pg/mL, n = 524), group 3 (serum E_2_ levels 2001–3000 pg/mL, n = 783), group 4 (serum E_2_ levels 3001–4000 pg/mL, n = 721), group 5 (serum E_2_ levels 4001–5000 pg/mL, n = 548), and group 6 (serum E_2_ levels > 5000 pg/mL, n = 852)^9^.

### Controlled hyper-stimulation induction and embryo transfer

All patients received one of the following four controlled ovarian stimulation (COS) regimens, which have been described previously^10^: gonadotropin-releasing hormone (GnRH)-agonist short protocol, GnRH-antagonist, mild stimulation and progestin-primed ovarian stimulation (PPOS). The clinician selected the appropriate protocol for each patient on an individual basis according to the patient characteristics.

Pituitary suppression was achieved by injecting triptorelin acetate (Decapeptyl 0.1 mg [Ferring, Germany] or Diphereline 0.1/3.75 mg [Ipsen, France]) until the serum levels of E2, follicle-stimulating hormone (FSH) and luteinizing hormone (LH) were < 30 mIU/mL, < 5 mIU/mL and < 5 mIU/mL, respectively. COS was initiated with several types of gonadotropin, namely, FSH (Gonal-F 75 IU[Serono, Switzerland], Fostiman 75 IU [IBSA, Switzerland],or Puregon 50 IU [N. V. Organon, Netherlands]) or FSH combined with LH (hMG, Livzon, China). In general, the initial dose of gonadotropin was determined according to the individual's age, BMI, basal follicle-stimulating hormone (bFSH) level, and response to previous stimulation cycles, as well as whether polycystic ovary syndrome (PCOS) was present^11^.

After stimulation for at least 4 days, the dose was adjusted according to the ovarian response, which was assessed using ultrasound and serum E2 measurements. When more than 3 follicles reached 17 mm, hCG was injected, and oocytes were extracted 36–37 h later^11^.

### Serum hormone measurements

bFSH was measured on days 2–4 of the menstrual cycle and prior to the start of the IVF cycle. In each cycle, LH, E2 and P levels were measured during controlled ovulation induction (once every 2–4 days at the beginning, once every day in the late follicular stage, and once on the day of hCG injection), and the gonadotropin and gonadotropin levels of each group were compared. All blood samples were obtained in the fasting state, usually between 6:30 and 7:30 am. Throughout the study, we used a personal immune analyser (Roche Cobas e411; Roche Diagnostics, Mannheim, Germany) and the same analytical methods were used to measure all hormones^11^.

### Data collection

With a standardized questionnaire telephone survey, specially trained nurses in our department conducted surveys of couples during each pregnancy to collect information on pregnancy complications, pregnancy date, place of birth, delivery mode, sex of the new-born, gestational age, birth weight, and neonatal diseases. In cases where attempts to contact the couple failed, the local family planning service was contacted to collect data.

### Outcome measures

The outcomes were birth weight indicators, including absolute birth weight and LBW. LBW was defined as birthweight < 2500 g. Preterm birth (PTB) was defined as delivery before 37 weeks of gestation. Based on a set of general population reference values for Chinese singletons^12^, Z-score was adopted for the standardization of birthweight after adjusting for gestational weeks and neonatal sex.

### Statistical analysis

All continuous variables are expressed as means ± SDs and categorical variables as frequencies or percentages. To examine significant differences among groups, the Mann–Whitney and chi-square tests were used for continuous variables and categorical variables, respectively. Multiple logistic regression models were applied to examine the relationship between serum E2 levels and neonatal birthweight. The relationship between the serum E2 level and neonatal birthweight was also explored using smoothing plots. A two-piecewise linear regression model was applied to investigate the threshold effect according to the smoothing plot. To quantify the strength of the association, unadjusted and adjusted odds ratios (ORs) and 95% confidence intervals (Cis) were estimated and reported. In the multivariate adjusted logistic regression models, the following were considered confounders: maternal age (years); sterility classification; duration of infertility (years); maternal BMI (kg/m^2^); basal FSH (mIU/mL); basal E2 (pg/mL); basal LH (mIU/mL); previous IVF attempts; ovarian stimulation protocol; total hMG dose (IU); duration of stimulation (days); number of oocytes retrieved; number of viable embryos; number of embryos transferred; gestational age at delivery (weeks); and neonatal sex. Interaction and stratified analyses included maternal age (years), maternal BMI, previous IVF attempts, and number of embryos transferred. Receiver operator characteristics (ROC) curves were constructed to identify the E2 threshold, and the corresponding area under the curve (AUC) was calculated.

Data were analysed with the use of the statistical packages R (The R Foundation; http://www.r-project.org; version 3.4.3) and EmpowerStats (www.empowerstats.com; X&Y Solutions, Inc., Boston, MA).

## Results

Overall, 3659 singleton live births from IVF cycles were included in this retrospective study. The numbers of patients with a peak serum E2 level of < 1000, 1000–1999, 2000–2999, 3000–3999, 4000–4999 and ≥ 5000 pg/mL were 230 (6.3%), 524 (14.3%), 783 (21.4%), 721 (19.7%), 548 (15.0%) and 852 (23.3%), respectively.

Table 1 shows the baseline demographic and periodic characteristics of the study cohort. Briefly, there were no significant differences in average age or BMI between the six groups, the average age and BMI of the first group were the highest, at 32.1 ± 4.4 years and 23.2 ± 3.2 kg/m^2^, respectively. The lowest hMG dose was 1939.5 ± 849.9 IU in the sixth group with the highest E2 level on the injection day of hCG. This may indicate that the low injection amount of hCG during downregulation may lead to a high E2 level on the hCG injection day. The majority of patients underwent double and cleavage-stage embryo transfer.

**Table 1:** Baseline characteristics of the study participants.

Baseline characteristics	Oestradiol level (pg/mL) on the day of trigger					
	≤ 1000	> 1000, ≤ 2000	> 2000, ≤ 3000	> 3000, ≤ 4000	> 4000, ≤ 5000	> 5000
N	230 (6.3%)	524 (14.3%)	783 (21.4%)	721 (19.7%)	548 (15.0%)	852 (23.3%)
Maternal age (years)	32.1 ± 4.4	31.8 ± 4.7	31.3 ± 4.4	30.9 ± 4.4	30.5 ± 4.6	30.1 ± 4.2
Duration of infertility (years)	4.2 ± 3.1	4.3 ± 3.3	4.1 ± 3.0	4.0 ± 2.9	4.0 ± 3.1	4.1 ± 3.0
Maternal BMI ( kg/m^2^)	23.2 ± 3.2	23.2 ± 3.2	22.8 ± 3.4	22.3 ± 3.0	22.0 ± 2.9	21.9 ± 2.9
Basal FSH (mIU/mL)	7.9 ± 4.8	7.7 ± 3.8	7.1 ± 2.2	7.1 ± 2.4	6.9 ± 2.0	6.9 ± 2.3
Basal E2 (pg/mL)	49.6 ± 59.2	47.2 ± 44.5	45.1 ± 38.7	47.8 ± 51.7	46.7 ± 45.2	45.3 ± 27.2
Basal LH (mIU/mL)	5.3 ± 4.0	4.9 ± 3.4	4.9 ± 2.8	5.2 ± 3.0	5.3 ± 2.9	5.8 ± 3.6
Total hMG dose (IU)	2751.3 ± 1308.9	2761.1 ± 1142.7	2593.5 ± 1014.4	2360.5 ± 972.7	2112.3 ± 848.9	1939.5 ± 849.9
Duration of stimulation (days)	10.8 ± 5.4	11.4 ± 4.7	11.3 ± 4.9	11.0 ± 4.8	10.3 ± 4.9	9.8 ± 5.2
number of oocytes retrieved	5.9 ± 5.3	6.4 ± 4.1	8.4 ± 4.6	9.1 ± 4.7	10.7 ± 5.2	12.6 ± 5.4
delivery gestational age (weeks)	38.6 ± 1.5	38.7 ± 1.5	38.9 ± 1.5	38.8 ± 1.5	38.9 ± 1.5	39.0 ± 1.4
Birthweight (g)	3496.7 ± 721.9	3456.8 ± 657.1	3451.1 ± 557.6	3443.3 ± 620.8	3407.8 ± 583.4	3496.9 ± 724.0
**Sterility classification**						
Primary infertility	112 (48.7%)	254 (48.5%)	361 (46.1%)	371 (51.5%)	279 (50.9%)	439 (51.5%)
Second infertility	118 (51.3%)	270 (51.5%)	422 (53.9%)	350 (48.5%)	269 (49.1%)	413 (48.5%)
**Previous IVF attempts**						
0	178 (77.4%)	439 (83.8%)	659 (84.2%)	630 (87.4%)	494 (90.1%)	777 (91.2%)
1	36 (15.7%)	60 (11.5%)	99 (12.6%)	76 (10.5%)	47 (8.6%)	60 (7.0%)
2	15 (6.5%)	17 (3.2%)	17 (2.2%)	11 (1.5%)	5 (0.9%)	12 (1.4%)
3	1 (0.4%)	6 (1.1%)	6 (0.8%)	4 (0.6%)	1 (0.2%)	3 (0.4%)
4	0 (0.0%)	1 (0.2%)	1 (0.1%)	0 (0.0%)	1 (0.2%)	0 (0.0%)
6	0 (0.0%)	1 (0.2%)	1 (0.1%)	0 (0.0%)	0 (0.0%)	0 (0.0%)
**Ovarian stimulation protocol**						
GnRH-a long protocol	102 (44.3%)	238 (45.4%)	366 (46.7%)	474 (65.7%)	416 (75.9%)	718 (84.3%)
GnRH-a prolonged (modified) protocol	111 (48.3%)	278 (53.1%)	413 (52.7%)	247 (34.3%)	131 (23.9%)	130 (15.3%)
mild stimulation	17 (7.4%)	8 (1.5%)	2 (0.3%)	0 (0.0%)	1 (0.2%)	1 (0.1%)
GnRH-agonist short protocol	0 (0.0%)	0 (0.0%)	2 (0.3%)	0 (0.0%)	0 (0.0%)	3 (0.4%)
**Number of embryos transfer**						
1	55 (23.9%)	52 (9.9%)	30 (3.8%)	17 (2.4%)	9 (1.6%)	20 (2.3%)
2	169 (73.5%)	446 (85.1%)	687 (87.7%)	648 (89.9%)	493 (90.0%)	764 (89.7%)
3	6 (2.6%)	26 (5.0%)	66 (8.4%)	56 (7.8%)	46 (8.4%)	68 (8.0%)
**Embryo stage at transfer, n (%)**						
Cleavage stage Blastocyst stage	193(83.9%) 37(16.1%)	433(82.6%) 91(17.4%)	651(83.1%) 132(16.9%)	601(83.4%) 120(16.6%)	450(82.1%) 98(17.9%)	712(83.6%) 140(16.4%)

Data are presented as the mean ± SD or number (percentage). BMI: Body mass index; FSH: Follicle-stimulating hormone; E2: Oestradiol; LH: Luteinizing hormone; IVF: In vitro fertilization.

Table 2 shows the single-factor analysis of neonatal birthweight. As shown, maternal BMI (kg/m^2^), basal LH (mIU/mL), and gestational age at delivery (weeks) were strongly correlated with neonatal birthweight (all *P*-values < 0.001). The increase in maternal gestational age is positively correlated with neonatal weight, which is consistent with normal physiological dynamics. However, there seems to be no similar study on whether maternal serum basal LH is associated with neonatal birth weight, so further statistical analysis of the clinical sample data or basic experiments are needed to verify this. In addition, the effect of different ovulation induction schemes for fresh cycles on new-born birthweight was also correlated, the specific mechanism remains to be explored.

**Table 2:** Univariate analysis of neonatal birthweight.

Comparison	Statistics	Sig	Exp(B)	95% CI	
				Lower	Upper
Maternal age (years)	30.9 ± 4.5	0.071	4.3	− 0.4	8.9
**Sterility classification**					
Primary infertility	1816 (49.6%)	0			
Second infertility	1842 (50.4%)	0.458	15.7	− 25.8	57.2
Duration of infertility (years)	4.1 ± 3.0	0.065	6.5	− 0.4	13.3
**Previous IVF attempts**					
0	3177 (86.9%)	0			
1	378 (10.3%)	0.675	14.6	− 53.7	83.0
2	77 (2.1%)	0.898	− 9.5	− 154.3	135.4
≥ 3	26 (0.7%)	0.628	61.2	− 186.1	308.6
Maternal BMI ( kg/m^2^)	22.5 ± 3.1	< 0.001	20.3	13.7	27.0
Basal FSH (mIU/mL)	7.2 ± 2.8	0.101	6.3	− 1.2	13.8
Basal E2 (pg/mL)	46.5 ± 42.8	0.673	− 0.1	− 0.6	0.4
Basal LH (mIU/mL)	5.2 ± 3.2	< 0.001	11.2	4.8	17.7
**Ovarian stimulation protocol**					
GnRH-a long protocol	2314 (63.3%)	0			
GnRH-a prolonged (modified) protocol	1310 (35.8%)	< 0.001	− 99.3	− 142.6	− 56.0
Mild stimulation	29 (0.8%)	0.285	− 127.6	− 361.5	106.3
GnRH-agonist short protocol	5 (0.1%)	0.018	676.9	116.5	1237.3
Total hMG dose (IU)	2357.1 ± 1034.9	0.069	0.0	− 0.0	0.0
Duration of stimulation (days)	10.7 ± 5.0	0.111	− 3.4	− 7.5	0.8
Serum E2 (pg/ml), hCG day	3736.4 ± 2123.3	0.154	0.0	− 0.0	0.0
Number of oocytes retrieved	9.4 ± 5.4	0.369	− 1.8	− 5.6	2.1
**Number of embryos transferred**					
1	183 (5.0%)	0			
2	3207 (87.7%)	0.279	− 52.6	− 147.9	42.7
3	268 (7.3%)	0.130	93.0	− 27.3	213.2
Gestational age at delivery (weeks)	38.9 ± 1.5	< 0.001	117.7	104.2	131.2

Outcome: Birthweight (g).

Exposure: Maternal age (years); Sterility classification; Duration of infertility (years); Previous IVF attempts NEW; Maternal BMI (kg/m^2^); Basal FSH (mIU/mL); Basal E2 (pg/mL); Basal LH (mIU/mL); Ovarian stimulation protocol; Total hMG dose (IU); Duration of stimulation (days); Serum E2 (pg/ml), hCG day; Number of oocytes retrieved; Number of viable embryos; Number of embryos transferred; Gestational age at delivery (weeks);

Serum E2 (pg/ml), hCG day; Oestradiol level (pg/mL) on trigger day.

**Figure 1:**
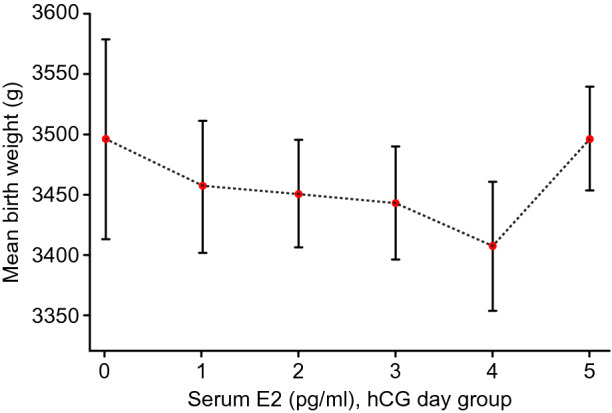
Relationship between serum E2 levels and neonatal birthweight.

Table 3 shows the neonatal outcomes grouped by oestradiol level on the trigger day. The average gestational age at delivery in the group 6 (serum E2 levels > 5000 pg/mL) was 39.0 ± 1.4 weeks (*P* = 0.010). The lowest mean neonatal birthweight in group 5 (serum E2 levels 4001–5000 pg/mL) was 3407.8 ± 583.4 g (*P* = 0.167). This is consistent with the line graph of the mean after adjustments for variables in Fig. 1. The mean new-born birthweight in the group 5 showed a slight downward trend. In addition, the LBW rates in the six groups were 3.0%, 2.9%, 1.9%, 2.9%, 2.9%, and 2.0% (*P* = 0.629), respectively. There were no statistically significant differences in the incidences of neonatal LBW among the six groups *(P* = 0.629). At the same time, there is no significant statistical difference in the Z-score between the six groups(*P* = 0.345). In view of this contradiction, we continued to carry out data analysis.

**Table 3:** Neonatal outcomes grouped by oestradiol level on trigger day.

Baseline characteristics	Grouped by oestradiol level (pg/ml) on the day of trigger						
	< = 1000	> 1000, < = 2000	> 2000, < = 3000	> 3000, < = 4000	> 4000, < = 5000	> 5000	*P*-value
N	230	524	783	721	548	852	
Delivery gestational age (weeks)	38.6 ± 1.5	38.7 ± 1.5	38.9 ± 1.5	38.8 ± 1.5	38.9 ± 1.5	39.0 ± 1.4	0.010*
Preterm birth, n (%)	13 (5.7%)	29 (5.6%)	35 (4.5%)	36 (5.0%)	20 (3.7%)	30 (3.5%)	0.377
Birthweight (g)	3496.7 ± 721.9	3456.8 ± 657.1	3451.1 ± 557.6	3443.3 ± 620.8	3407.8 ± 583.4	3496.9 ± 724.0	0.167
Sex							0.783
Male	124 (53.9%)	257 (49.0%)	400 (51.1%)	375 (52.0%)	290 (52.9%)	444 (52.1%)	
Female	106 (46.1%)	267 (51.0%)	383 (48.9%)	346 (48.0%)	258 (47.1%)	408 (47.9%)	
Birthweight (g) group							0.629
≥ 2500 (Non-LBW)	223 (97.0%)	509 (97.1%)	768 (98.1%)	700 (97.1%)	532 (97.1%)	835 (98.0%)	
< 2500 (LBW)	7 (3.0%)	15 (2.9%)	15 (1.9%)	21 (2.9%)	16 (2.9%)	17 (2.0%)	
Z-score	0.42 ± 1.00	-0.39 ± 1.11	-0.40 ± 1.05	-0.42 ± 1.07	-0.37 ± 1.02	0.36 ± 1.02	0.345

Data are presented as the mean ± SD or number (percentage).

LBW: low birth weight.

Non-LBW: non-low birth weight.

*P*-values < 0.05 indicate significant differences and are bolded.

In Table 4, we conducted a stratified analysis of neonatal birthweight. The statistical results showed that the previous conclusions were stable and reliable, and there was no statistically significant difference in neonatal weight among the six groups. At the same time, in Table 5, we adjusted for confounders and conducted a multiple regression analysis of intergroup LBW outcomes, the results of which supported our conclusions above.

**Table 4:** Geometric mean values for neonatal birthweight (g) across categories.

	Grouped by oestradiol level (pg/ml) on the trigger day																					
Y = Birthweight (g)	N	≤ 1000	> 1000, ≤ 2000				> 2000, ≤ 3000				> 3000, ≤ 4000				> 4000, ≤ 5000				> 5000			
			Sig	Exp(B)	95% CI		Sig	Exp(B)	95% CI		Sig	Exp(B)	95% CI		Sig	Exp(B)	95% CI		Sig	Exp(B)	95% CI	
					Lower	Upper			Lower	Upper			Lower	Upper			Lower	Upper			Lower	Upper
**Maternal age (years) group**																						
Group 1 (< 26.5)	887	0	0.286	121.7	− 101.7	345.0	0.763	33.0	− 181.1	247.2	0.288	115.2	− 96.9	327.2	0.436	85.9	− 130.3	302.1	0.550	63.3	− 144.2	270.8
Group 2 (≥ 26.5, < 29.1)	909	0	0.007*	− **274.1**	− **471.1**	− **77.1**	0.024*	− **211.3**	− **393.9**	− **28.7**	0.012*	− **237.6**	− **423.1**	− **52.1**	0.013*	− **239.1**	− **427.0**	− **51.2**	0.132	− 137.8	− 317.1	41.4
Group 3 (≥ 29.1, < 31.7)	816	0	0.185	143.0	− 68.1	354.0	0.077*	**179.0**	− **19.2**	**377.1**	0.407	84.8	− 115.4	284.9	0.896	− 14.2	− 227.1	198.7	0.132	151.4	− 45.2	348.1
Group 4 (≥ 31.7)	1046	0	0.582	− 50.0	− 228.0	128.0	0.367	− 79.0	− 250.7	92.6	0.439	− 69.7	− 246.1	106.7	0.311	− 96.7	− 283.7	90.2	0.491	63.1	− 116.5	242.6
**Maternal BMI ( kg/m**^**2**^**) group**																						
Group 1 (< 15.75)	527	0	0.052	− 288.7	− 579.3	1.9	0.022*	− **303.3**	− **562.3**	− **44.3**	0.007*	− **355.5**	− **613.1**	− **97.8**	0.005*	− **378.0**	− **639.4**	− **116.6**	0.166	− 176.7	− 426.6	73.2
Group 2 (≥ 15.75, < 21.25)	1045	0	0.602	− 53.1	− 252.4	146.2	0.284	− 105.1	− 297.5	87.3	0.761	− 29.5	− 219.5	160.4	0.652	− 45.1	− 240.9	150.7	0.635	− 45.2	− 231.8	141.5
Group 3 (≥ 21.25, < 26.75)	897	0	0.750	33.6	− 173.2	240.5	0.556	58.4	− 135.9	252.7	0.950	6.3	− 192.3	204.8	0.548	61.7	− 139.6	263.0	0.204	125.2	− 67.7	318.1
Group 4 (≥ 26.75)	1189	0	0.981	− 1.9	− 159.2	155.4	0.765	23.0	− 127.7	173.8	0.690	31.7	− 124.1	187.6	0.281	− 93.2	− 262.8	76.3	0.397	68.1	− 89.5	225.6
**Sex**																						
Male	1890	0	0.529	− 42.9	− 176.7	90.9	0.411	− 52.8	− 178.6	73.0	0.128	− 98.5	− 225.2	28.3	0.048*	− **132.4**	− **263.7**	− **1.1**	0.811	− 15.2	− 139.5	109.1
Female	1768	0	0.745	− 24.4	− 171.9	123.0	0.672	− 30.4	− 171.4	110.5	0.992	0.8	− 141.8	143.4	0.622	− 37.2	− 185.4	110.9	0.759	21.9	− 118.1	161.9
**Number of embryos transferred**																						
1	183	0	0.098*	− **229.3**	− **499.3**	**40.6**	0.411	− 133.2	− 449.9	183.6	0.191	− 259.5	− 646.8	127.8	0.720	− 91.8	− 593.7	410.0	0.313	188.2	− 176.2	552.6
2	3207	0	0.904	− 6.6	− 113.1	100.0	0.797	− 13.3	− 114.6	88.0	0.659	− 23.0	− 124.9	78.9	0.401	− 45.1	− 150.3	60.1	0.819	11.7	− 88.6	112.0
3	268	0	0.980	10.6	− 832.8	853.9	0.585	− 221.4	− 1015.4	572.6	0.692	− 161.6	− 961.5	638.3	0.331	− 401.3	− 1209.6	406.9	0.968	− 16.2	− 809.2	776.8

*P*-values < 0.05 indicate significant differences and are bolded.

**Table 5:** Multivariable logistic model for all patients grouped by oestradiol level on the trigger day.

Oestradiol level (pg/ml) on the trigger day	Non-adjusted				Adjustment I				Adjustment II			
	Sig	Exp(B)	95% CI		Sig	Exp(B)	95% CI		Sig	Exp(B)	95% CI	
			Lower	Upper			Lower	Upper			Lower	Upper
≤ 1000	–	1.0	–	–	–	1.0	–	–	–	1.0	–	–
> 1000, ≤ 2000	0.892	0.9	0.4	2.3	0.905	0.9	0.4	2.4	0.905	1.1	0.3	3.3
> 2000, ≤ 3000	0.307	0.6	0.3	1.5	0.325	0.6	0.3	1.6	0.615	0.7	0.2	2.4
> 3000, ≤ 4000	0.919	1.0	0.4	2.3	0.949	1.0	0.4	2.3	0.663	1.3	0.4	4.1
> 4000, ≤ 5000	0.926	1.0	0.4	2.4	0.968	1.0	0.4	2.4	0.437	1.6	0.5	5.5
> 5000	0.342	0.6	0.3	1.6	0.383	0.7	0.3	1.6	0.625	1.4	0.4	4.8

Non-adjusted model: no adjustments.

Adjustment model I: adjusted for maternal age (years); sterility classification; duration of infertility (years); and maternal BMI (kg/m^2^).

Adjustment model II: adjusted for maternal age (years); sterility classification; duration of infertility (years); maternal BMI (kg/m^2^); basal FSH (mIU/mL); basal E2 (pg/mL); basal LH (mIU/mL); previous IVF attempts; ovarian stimulation protocol; total hMG dose (IU); duration of stimulation (days); number of viable embryos; number of embryos transferred; Stage of embryo transfer; gestational age at delivery (weeks); and neonatal sex.

**Figure 2:**
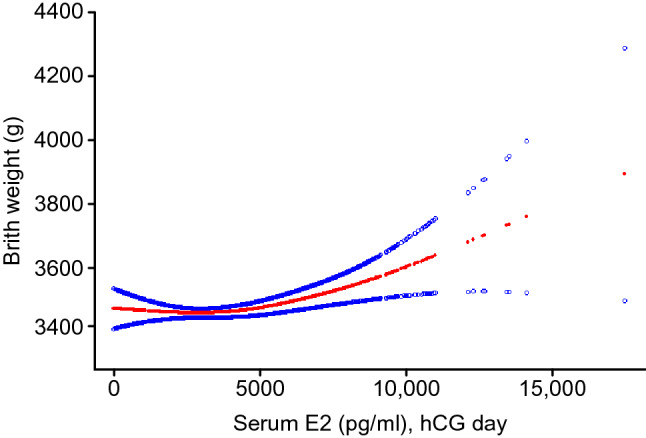
The relationship between serum E2 levels on the trigger day and neonatal birthweight. A nonlinear relationship between serum E2 levels and neonatal birthweight was observed after adjusting for maternal age, maternal BMI, number of embryos transferred, neonatal sex.

**Figure 3:**
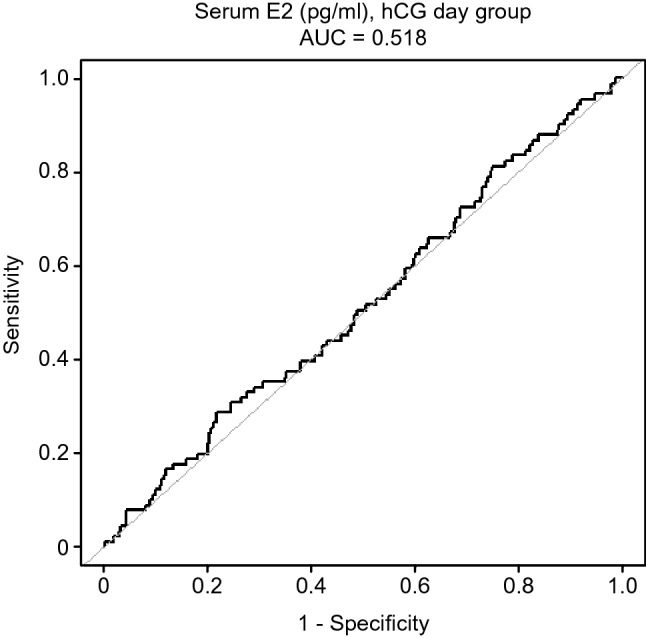
Receiver operating characteristic (ROC) curve evaluation of the ability of peak E2 measurements to predict low birth weight. The best threshold is 2062 pg/ml with a sensitivity of 28.6%, a specificity of 78.1%, and an area under the curve of 0.518.

To explore the relationship between serum E2 level and neonatal weight on hCG day and whether there is a threshold effect, the analyses shown in Figs. 2 and 3 were performed. Figure 2 shows a nonlinear relationship between serum E2 levels and neonatal birthweight after adjusting for maternal age, maternal BMI, number of embryos transferred, and neonatal sex. In Fig. 3**,** the ROC curve is presented to evaluate the ability of peak E2 measurements to predict LBW. The best threshold is 2062 pg/ml with a sensitivity of 28.6%, a specificity of 78.1%, and an AUC of 0.518. Such a small AUC value does not have sufficient strength as evidence.

## Discussion

The results of this retrospective cohort study showed that serum E2 peak levels during ovarian stimulation were not associated with birthweight during IVF cycles. In addition, there was no association between higher E2 levels and increased LBW risk. Our observations suggest that the hyper-oestrogenic milieu during COS does not seem to have adverse effects on the birthweight of offspring after IVF.

A supraphysiologic E2 level is inevitable during COH, and its effect on the outcome of IVF-ET has been controversial. Oestrogen and its receptors are a major factor in improving endometrial receptivity to initiate embryo implantation^13^. There is increasing evidence that hyper-physiological E2 levels during COS may lead to decreased endometrial and sub-endometrial blood flow^14^. These harmful effects not only impair early embryo adhesion and implantation^15^ but also harm placental formation and subsequent foetal growth^16^. The high oestradiol group had a high incidence of pregnancy complications associated with placental abnormalities, including foetal growth restriction, pregnancy-related hypertension, and abnormal placental implantation^16^. Therefore, in clinical work, the possible risk of OHSS and the pregnancy complications associated with placental abnormalities should be considered when deciding on embryo transfer in high responders. However, the results of our retrospective cohort study showed that serum E2 peak levels during ovarian stimulation were not associated with birthweight during IVF cycles. Zavy et al.^6^ and Wang et al.^7^ reported that an elevated serum E2 level on the day of hCG injection does not change the pregnancy rate, indirectly confirming our conclusion.

Recently, Imudia et al.^17^ found that the fertilization rate of normal oocytes, the embryo development rate and the positive rate for pregnancy showed an overall decreasing trend with increasing oestradiol level^17^. However, Imudia et al.^17^ found that a serum E2 level that exceeds the 90th percentile on hCG injection day is associated with a decreased oocyte fertilization rate but that this level does not affect embryo development, the implantation rate, the clinical pregnancy rate or spontaneous abortion rate^17^. Chen et al.^18^ showed that peak E2 levels do not adversely affect treatment outcomes. Wu et al.^19^ suggested that a single-day high E2 concentration on the hCG trigger day would not affect the pregnancy outcome; however, when combined with a premature progesterone elevation, this high E2 concentration may have adverse effects on pregnancy outcomes. For these patients, frozen-thawed embryo transfer (FET) should be recommended to improve pregnancy outcomes^19^. Other studies^19–21^ have reported similar results in response to the view that elevated E2 levels do not have any harmful effects on pregnancy rates.

Understanding and managing patients with ovarian hyper-responsiveness in clinical practice can be a challenge for physicians. In our study, patients with OHSS were excluded. In addition to OHSS, hyper-physiological levels of oestradiol are well documented and may cause endometria-embryo dys-synchrony, which may have a negative impact on pregnancy rates. After controlling for embryo quality, Zavy et al.^6^ showed that the live birth rate is not affected by the increase in E2 level on hCG injection day, and this conclusion is consistent with the actual practice in our clinical work, as well as our statistical conclusion. In our clinical work, high-quality embryos are given priority for transplantation. This may lead to a statistical bias in selectivity. However, this is more in line with the actual situation in clinical work. Our study showed that, after controlling for embryo quality, an increase in oestradiol levels on the day of hCG injection was neither directly harmful nor beneficial to neonatal birthweight. At the same time, our data suggest that high-quality embryos may be able to tolerate suboptimal uterine environments that have been exposed to hyper-physiological levels of oestradiol. Therefore, the quality control of embryos is a very important part of IVF.

Finally, this study still has some limitations. After successful pregnancy, the perinatal care was transferred to obstetrics in different hospitals. The follow-up of patients is obtained through telephone interviews by professionally trained nurses of our center. This is different from directly consulting the patient's medical record system, and there may be some data errors. At the same time, due to the inability to share electronic medical records of patients in different hospitals, other data related to perinatal outcomes cannot be obtained. Such limitations will indeed cause the problem of selection bias, which is not good for a more comprehensive discussion of the factors affecting neonatal outcomes. Second, our database lacks detailed embryo quality grades, which is also an important confounding factor for neonatal outcomes. In the future data collection process, we will strengthen the improvement of data types.

## Conclusion

In clinical work, controlling the quality of transplanted embryos is very important. We did not detect an association between peak serum E2 level during ovarian stimulation and neonatal birthweight IVF-ET cycles. This finding provides reliable information not only for patients with high ovarian response but also for clinicians in choosing an appropriate COH regime.

## Abbreviations

hCG: Human chorionic gonadotrophin
IVF-ET: In vitro fertilization-embryo transfer
BMI: Body mass index
FSH: Follicle-stimulating hormone
E2: Oestradiol
LH: Luteinizing hormone
IVF: In vitro fertilization
LBW: Low birth weight
Non-LBW: Non-low birth weight.
OHSS: Ovarian Hyper-stimulation Syndrome
